# Ending Neglected Surgical Diseases (NSDs): Definitions, Strategies, and Goals for the Next Decade

**DOI:** 10.34172/ijhpm.2020.140

**Published:** 2020-08-11

**Authors:** Jaymie A. Henry, Angela S. Volk, Sicily K. Kariuki, Kiraitu Murungi, Trina Firmalo, Ruth Laibon Masha, Orion Henry, Peter Arimi, Patrick Mwai, Estella Waiguru, Evans Mwiti, Dan Okoro, Angella Langat, Cosmas Mugambi, Erin Anastasi, Gillian Slinger, Chris Lavy, Rosalind Owen, Erin Stieber, Marc Lester Suntay, Danny Haddad, Robert Lane, Joel Buenaventura, Neil Parsan, Fizan Abdullah, Michael Nebeker, Lismore Nebeker, Charles Mock, Larry Hollier, Pankaj Jani

**Affiliations:** ^1^The Global Alliance for Surgical, Obstetric, Trauma, and Anesthesia Care (G4 Alliance), Chicago, IL, USA.; ^2^International Collaboration for Essential Surgery (ICES), Boca Raton, FL, USA.; ^3^Department of Surgery, Florida Atlantic University (FAU), Boca Raton, FL, USA.; ^4^Baylor College of Medicine Division of Plastic Surgery, Texas Children’s Hospital, Houston, TX, USA.; ^5^Ministry of Health Kenya, Nairobi, Kenya.; ^6^County Government of Meru County, Meru County, Kenya.; ^7^Provincial Government of Odiongan, Odiongan, Philippines.; ^8^Joint United Nations Programme on HIV/AIDS (UNAIDS), Geneva, Switzerland.; ^9^Finders Keepers Technologies LLC, Boca Raton, FL, USA.; ^10^University of Nairobi College of Health Sciences, Nairobi, Kenya.; ^11^Mount Kenya University (MKU), Thika, Kenya.; ^12^United Nations Population Fund (UNFPA), Nairobi, Kenya.; ^13^Beyond Zero Secretariat, Kenya First Ladies’ Office, Nairobi, Kenya.; ^14^United Nations Population Fund (UNFPA), Campaign to End Fistula, New York City, NY, USA.; ^15^International Federation of Gynecology and Obstetrics (FIGO), Vancouver, BC, Canada.; ^16^University of Oxford, Oxford, UK.; ^17^Global Clubfoot Initiative (GCI), London, UK.; ^18^Smile Train International, New York City, NY, USA.; ^19^World Surgical Foundation (WSF) Philippines, Manila, Philippines.; ^20^Orbis International, New York City, NY, USA.; ^21^International Federation of Surgical Colleges (IFSC), London, UK.; ^22^Child Family Health International Philippines, Manila, Philippines.; ^23^Government of Trinidad and Tobago, Port of Spain, Trinidad and Tobago.; ^24^Northwestern University Lurie Children’s Hospital, Chicago, IL, USA.; ^25^Mobile Surgery International, Oaxaca, Mexico.; ^26^University of Washington Harborview Injury Prevention and Research Center, Seattle, WA, USA.; ^27^College of Surgeons of East, Central, and Southern Africa (COSECSA), Arusha, Tanzania.

**Keywords:** Neglected Surgical Diseases, NSD, Neglected Surgical Conditions, Untreated Surgical Conditions, Essential Surgery, Global Surgery

## Abstract

While there has been overall progress in addressing the lack of access to surgical care worldwide, untreated surgical conditions in developing countries remain an underprioritized issue. Significant backlogs of advanced surgical disease called neglected surgical diseases (NSDs) result from massive disparities in access to quality surgical care. We aim to discuss a framework for a public health rights-based initiative designed to prevent and eliminate the backlog of NSDs in developing countries. We defined NSDs and set forth six criteria that focused on the applicability and practicality of implementing a program designed to eradicate the backlog of six target NSDs from the list of 44 Disease Control Priorities 3rd edition (DCP3) surgical interventions. The human rights-based approach (HRBA) was used to clarify NSDs role within global health. Literature reviews were conducted to ascertain the global disease burden, estimated global backlog, average cost per treatment, disability-adjusted life-years (DALYs) averted from the treatment, return on investment, and potential gain and economic impact of the NSDs identified. Six index NSDs were identified, including neglected cleft lips and palate, clubfoot, cataracts, hernias and hydroceles, injuries, and obstetric fistula. Global definitions were proposed as a starting point towards the prevention and elimination of the backlog of NSDs. Defining a subset of neglected surgical conditions that illustrates society’s role and responsibility in addressing them provides a framework through the HRBA lens for its eventual eradication.

## Introduction

 Despite steady gains in raising the profile of surgery in public health, untreated surgical conditions continue to plague certain societies, causing 25% of avoidable mortality^1^ and up to 40% of avoidable morbidity^2,3^ in low- and middle-income countries (LMICs). Unaddressed, these conditions progress to profound disability, deformity, or early death due to a lack of timely access to essential surgical care. These conditions, highlighted by the World Bank’s *Disease Control Priorities* 3rd edition (DCP3) on Essential Surgery,^4^ remain the poster children of surgical neglect. Previous articles have called for greater attention to these conditions,^5-8^ yet no current global strategy exists to systematically end the neglect. In this perspective, we will expound and discuss the concept of ‘neglected surgical diseases,’ or NSDs. We define NSDs as “any treatable surgical condition that has progressed beyond functional repair due to a lack of access to timely surgical care.” NSDs disproportionately impact citizens of LMICs, resulting in an enormous burden on society with lost economic productivity, social stigma, and immense suffering. Implementing safe and cost-effective interventions,^9^ treatment could avert an estimated 17 million deaths and over 77 million disability-adjusted life-years (DALYs) annually.^4^ However, due to stigma, lack of awareness, lack of access, political will, and financial support, these conditions are not systematically addressed in most public health programs or treated in primary care or community settings.

 An additional 143 million surgical procedures are required each year to meet the need in LMICs.^10^ Of those receiving surgery, high perioperative mortality and complication rates are seen.^11^ A review of National Health Strategic Plans in 43 sub-Saharan African countries revealed a lack of surgical prioritization: 19% had no mention of surgery and only 47% referenced traumatic conditions despite high injury mortality rates in those regions.^12^ Most NSDs are not included in national censuses or are underestimated in global health databases. In the worst affected countries, the political language for ‘Global Surgery’ has yet to be written.^13-16^ Yet an average of USD 573 million in aid are spent yearly on surgical conditions, mostly from non-governmental organizations (NGOs) run by high-income countries, signifying not only backlogs in underdeveloped regions,^11^ but also funding interest from the global health community.^17^ The question we now face is how to leverage these resources and build systems of care that are integrated, sustainable, dignified, inclusive, and more importantly, local, in order to leave a legacy of surgical capacity building in the most remote areas of need.

 As the world looks to achieve the Sustainable Development Goals by 2030 with a grand, but attainable vision for universal health coverage (UHC): ‘Leaving No One Behind;’ strategies to improve surgical access, availability, and safety for the bottom billion are paramount. Global progress has been achieved in advancing surgical care with the World Health Organization (WHO) resolution on Essential Surgery,^18^ the landmark publication of the Lancet Commission on Global Surgery (LCoGS)^10^, and current work on national surgical plans^19,20^; additional gains can be achieved with addressing NSDs complementing these efforts through a systematic approach that unifies the intersection of human rights, public health, and Global Surgery. We therefore aim to: (1) discuss an approach for classifying six NSDs from a human rights-based perspective, (2) propose a global definition of the neglect, and (3) propose a public health strategy for addressing these conditions at a global level.

## A Human Rights-Based Approach

 Health inequities are avoidable differences in health between population groups caused by socioeconomic conditions.^21^ The recent LCoGS report estimated that only 6% of all surgical procedures done worldwide occur in the poorest nations where a third of the global population reside,^10^ highlighting a massive issue of surgical inequity. Global backlogs, therefore, exist due to this disparity. Traditional frameworks have been proposed for addressing health inequities in general, which incorporate a patient-centered design including families and communities, data analytics, and continuous improvement.^22^ A human rights-based approach (HRBA) does not ‘simply describe the situation in terms of human needs, but also incorporates society’s obligation to respond to the inalienable rights of individuals and empowers people to demand justice as a right.’^23^ Thus, addressing NSDs with this approach clarifies its role within the global health milieu.^24^ The following HRBA principles were applied to clarifying the role of NSD in global health. In brief, *universality of protection* ensures that marginalized surgical patients should be included as part of primary care or UHC programs insofar as being able to utilize public resources to aid in screening, detection, reporting, treatment, and to offer financial protection when needed. Institutionalizing surgery within public health addresses HRBA’s principles of having *comprehensive, coherent and coordinated policies* for neglected surgical patients with adequate legal and institutional frameworks, long-term social protection strategies, standards of accessibility, adaptability and acceptability, and adequacy of benefits such as timely access to care. The principle of *dignity, autonomy, equality and non-discrimination* protects surgical patients’ right to public policies and services that reach the most marginalized. *Gender perspective* highlights surgery’s indispensable role in mitigating women’s morbidity and mortality from a lack of access to emergency obstetric surgical services. Formal involvement of local government agencies with active civil society participation in programs that address surgical inequities address the principles of *access to accountability mechanisms and effective remedies*. *Community involvement* in the prevention and detection of surgical conditions ensure meaningful and effective participation.

 For purposes of defining NSDs, a usable set of index surgical conditions for a target surgical population was generated from the DCP3 list of 44 surgical interventions using six criteria to address the applicability and practicality of implementing the NSD program focusing on key conditions:

- *Impact.* A candidate disease has an avertable high lifetime DALY burden.
- *Cost-effectiveness.* The cost of performing the surgery in a low-resource setting is much lower than the societal cost of DALYs carried by a disabled person.
- *Feasibility.* The surgical intervention can be done in a low-resource setting and do not require advanced equipment to be done safely.
- *Quality and safety.* The candidate disease is not an emergency, allowing a local government unit (LGU) district and/or referral hospital or its equivalent to ensure that they can deliver quality safe surgery before having to handle the complexities of safe emergency services.
- *Relevance.* The candidate disease forms part of a group of conditions that confer a significant burden of morbidity and mortality in a population of neglected patients large enough to ensure that local or specially designated providers can be trained and/or supported fully in the LGU district and/or referral hospital working on their local population.
- *Public health significance. *The candidate disease can be prevented or the backlog of neglected, advanced cases can be eradicated through early detection and management.

Criteria 1 and 2 disarm the argument that surgical care is too expensive, whereas NSDs, by definition, are too expensive *not* to treat.^9^ Criteria 3, 4, and 5 ensure that any LGU district and/or referral hospital can cost-effectively and easily upgrade their current facilities and train their current staff to deal with NSDs. Criterion 5 ensures that adequate training can be done on site at the LGU district and/or referral hospital, thus addressing concerns of “brain drain” and the difficulties of relocating surgeons to work in remote rural hospitals. These criteria emphasize a ‘whole of system’ approach in the prevention and elimination of NSDs, bringing the treatment close to the people as most of them are found in remote and under resourced communities.

 Based on these criteria, all emergent or time-dependent conditions and preventive surgical interventions were excluded as well as two other conditions that did not meet the definition of an NSD and >50% of the NSD criteria (excision of common benign tumors and repair of anorectal malformation and Hirschsprung’s disease). Six NSDs were therefore identified (Table 1) and discussed with proposed global definitions to establish uniformity and markers of progress (Table 2). The global disease burden, estimated global backlog, average cost per treatment, DALYs averted from the treatment, information on return on investment, and potential gain and economic impact from literature reviews are presented in Table 3.

**Table 1:** DCP3 Essential Surgery List of 44 Surgical Interventions That Meet the Predefined NSD Criteria

**DCP3 Non-urgent Conditions**	**Impact**	**Cost-Effectiveness**	**Feasibility**	**Quality and Safety**	**Relevance**	**Public Health Significance**
Fracture reduction (malunion)	x	x	x	x	x	x
Hydrocelectomy	x	x	x	x	x	x
Elective hernia repair	x	x	x	x	x	x
Excision of common benign tumors	-^a^	-^a^	x	x	-^a^	x
Cataract extraction and insertion of intraocular lens	x	x	x	x	x	x
Eyelid surgery for trachoma	x	x	x	x	x	x
Repair obstetric fistula	x	x	x	x	x	x
Repair of cleft lip and palate	x	x	x	x	x	x
Repair of anorectal malformations and Hirschsprung’s disease	x	-^b^	-^c^	-^d^	-^e^	x

Abbreviations: DCP3, Disease Control Priorities 3rd edition; NSD, neglected surgical disease. All emergent procedures excluded, a- variable, depends on location and size of tumor, prevalence difficult to determine due to heterogeneous nature of disease burden, b- will need more resources to treat, c- will need specially trained providers, d- emergency decompression needed at birth, e- relative numbers not large enough.

**Table 2:** Proposed Global Definition for Index NSDs

**NSDs**	**Proposed Global Definition**
Neglected cleft lip and/or palate	Cases of unrepaired CLP beyond 6 (lip) and 18 (palate) months of age 25 Surgical repair occurring below the disease incidence rate Lack of systems and comprehensive cleft care teams to identify and properly manage cleft disease using defined strategies, with appropriately available financial resources
Neglected clubfoot	Cases of unrepaired clubfoot beyond two years of age 26 Initiation of treatment at a rate below the reported incidence in LMICs Lack of systems and care teams to identify and properly manage clubfoot deformities using defined strategies, with appropriately available financial resources
Neglected avoidable blindness	Blindness caused by any preventable disease, especially unrepaired cataracts and trachoma Initiation of treatment at a rate below the reported incidence in LMICs Lack of access to proper surgical care, including a trained ophthalmic surgical provider, proper equipment and infrastructure, supporting teams, and available financial resources
Neglected injuries	Lack of accessible trauma care systems to provide life or limb-saving treatment during the appropriate window of time to prevent premature death or unnecessary disability Lack of legislation to improve road safety, lack of access to additional trauma-related care services, supporting teams, and available financial resources
Neglected obstetric fistula	Cases of unrepaired obstetric fistula for more than one year Lack of systems and enablers (both supply and demand-side) to ensure timely, quality care to identify and properly manage and prevent women from developing an obstetric fistula
Neglected hernia and/or hydrocele	Any unrepaired symptomatic or advanced groin hernia and/or hydrocele in LMIC populations Lack of systems and care teams to identify hernia and/or hydrocele presence with appropriately available infrastructures and financial resources to perform surgical repair

Abbreviations: CLP, cleft lip and/or palate; NSD, neglected surgical disease; LMIC, low- and middle-income country.

**Table 3:** NSD Estimated Global Prevalence, Incidence, Backlog, Cost of Treatment, DALY Averted, ROI, and Economic Impact of Treatment

	**CLP**	**Clubfoot**	**Cataracts**	**Injuries**	**Hernia**	**Fistula**
2017 GBD^15^ prevalence (thousands)	10 816.4 (9945.7 to 11 654.1)	16 262.8 (14 519.3 to 18 611.7) (other congenital musculo-skeletal anomalies)	107 987.7 (95 775.3 to 122 319.3)	1 507 481.4 (1 439 758.0 to 1 587 209.4)	26 490.8 (24 196.8 to 28 760.4) (inguinal, femoral, and abdominal hernia) LF: 64 623.4 (59 178.2 to 70 866.1)	1127.5 (939.1 to 1338.2) (maternal obstructed labour complications)
2017 GBD^15^ incidence (thousands)	195.5 (152.0 to 249.3)	929.0 (801.7 to 1074.0) (other congenital musculo-skeletal anomalies)	-^a^	520 710.3 (493 430.2 to 547 988.6)	41 182.8 (36 372.8 to 46 265.0), (inguinal, femoral, and abdominal hernia) 293 per 100 000 population	220.7 (179.2 to 267.7), (maternal obstructed labour complications) 50 000 to 100 000 worldwide develop a fistula annually^27,28^; 60 000 to 90 000 annually in SSA alone^27^
Estimated global backlog (or unmet need)	400 000 and 2 million globally^29^ Burden in LMIC (2014): 5000 deaths, 1 000 000 DALYS^27^	1 million (GCI) 72% of children going untreated each year^30^	20M (2010)^31^ Blindness: 12.6 M^32^ Blindness and MSVI: 65.2 M^32,33^	Burden in LMIC (2014): RTI: 1 160 000 deaths; 72 000 000 DALYS^27^ Other unintentional injuries: 1 550 000 deaths; 96 000 000 DALYS^27^ Intentional injuries: 540 000 deaths, 34 000 000 DALYS^27^	163-357 per 100 000 population per year for all cases^27^	Approximately 500 000 existing cases*
Average cost per treatment	US$250 per child US$10-110 per DALY averted^27^ Specialized cleft clinic in India: US$300 per DALY averted^34^	US$140-161 per child^35,36^ Cost per child will decrease over time from $317 in 2017 to $82 in 2030	Short-term cataract hospitals US$50 vs. NGO hospitals of $46 per case^27^ US$50 per DALY averted^27^	District trauma: $77-223/DALY averted^37,38^ Improving first aid skills of lay first responders <$10 per year of life gained^27^ Burn care: average daily cost per patient US$6; average overall cost for burn admission US$62^39^	Ecuador: $101 per DALY averted^27^ Ghana: $11 per DALY averted^27^ Inguinal hernia: US$10-100 per DALY averted^27^ Repair with mosquito-net mesh: US$12.88 per DALY averted^40^	US$400 per patient^27^ Emergency C-section: US$15-380 per DALY averted^27^ Emergency obstetric care in Bangladesh: $11 per DALY averted^41^
DALY averted	Estimated numberof avertable DALYs with scaling up surgical care: 5 076 572^27^	7.42 per case^36^	Estimated number of avertable DALYs with scaling up surgical care: 4 207 758^27^	Estimated number of deaths and DALYs averted per year that could be prevented if basic surgical care provided in LMIC: Deaths: 1 042 292 DALYs: 52 316 946^27^	Estimated number of deaths and DALYs averted per year that could be prevented if basic surgical care provided in LMIC: Deaths: 14 065 DALYs: 325 308^27^ 11 DALYs per 100 000 population^42^	Estimated number of deaths and DALY averted per year that could be prevented if basic surgical care provided in LMIC (obstructed labor): Deaths: 10 882 DALYs: 606 833^27^ Estimated number of avertable DALYs w/scaling up surgical care (fistula): 996 553^27^
ROI	$15.44-96.04 cost/DALY averted^9^ Benefit-cost ratio of 12 (specialized cleft clinic in India)^43^	Average cost-effectiveness ratio of $22.46 per DALY averted^36^ US$120 000 in regained lifetime earnings per child (or net present value of $13K per child treated)^35^	$5.06-$106 cost/DALY averted^9^	Emergency and trauma surgery cost/DALY averted $32.78-$223.00^9^ First level hospital in Bangladesh: US$11 per DALY averted^41^ First level hospital in Cambodia: US$78 per DALY averted^38^	$12.88-78.18 cost/DALY averted^9^	Emergency C-section $18-$3,462 cost/DALY averted^9^ C-section for obstructed labor: SSA: US$73 per DALY averted South-East Asia: US$2,638 per DALY averted^27^ C-section services vary widely by country from US$251 for each DALY averted in countries w/higher maternal morbidity risks to US$3462 per DALY averted in other countries^27,34^ Fistula surgery in Ethiopia – savings of US$40 per DALY averted^27^
Potential gain/ Economic Impact	$10 B in SSA alone^43,44^	1.2 million children in LMICs (70% of cases) were to be treated by 2030, US$1.5 billion in earning wound be generated^35^	Global economic impact of these treatable conditions has resulted in a productivity loss of US$19-168 billion and 25 million DALYs^27^	Trauma is the leading cause of death in men and women ages 15-44, the most economically productive population^45^	Annual combined economic loss due to disability from LF in India and SSA is estimated at US$1.5-2 billion	Analysis in India: combining cost savings of effective family planning with cost savings of providing emergency obstetric care could = savings of US$1.5B per year and reduction of maternal mortality by 75%^46^

Abbreviations: CLP, cleft lip and/or palate; MSVI, severe or moderate visual impairment; LMICs, low- and middle-income countries; GBD, Global Burden of Disease; LF, lymphatic filariasis; SSA, sub-Saharan Africa; GCI, Global Clubfoot Initiative; RTI, road traffic accidents; NGO, non-governmental organization; ROI; return on investment; NSD, neglected surgical disease; DALY, disability-adjusted life-year. * Total cost estimated from (estimated global backlog) (average cost per treatment). * United Nations Population Fund (UNFPA) Campaign to End Fistula/World Health Organization (WHO)/Johns Hopkins University 2020 updated estimates, unpublished data. ^a^ insufficient data.

## Disease Eradication: The Holy Grail of Public Health

 Unlike infectious diseases where eradication of a disease entity entails total abolition of a pathogen and its concomitant transmission pathways, surgical disease elimination confers a different mindset as surgical conditions arise from various etiologies – whether it be genetic, iatrogenic, functional, or environmental – and will arise regardless of the circumstance. What can be eliminated, however, are advanced forms of surgical disease that fundamentally incapacitates an individual and prevents that human being from living a humane and productive life as can be reasonably expected by society. This will entail ensuring the availability of timely access to surgical care which can be measured from a population perspective by: (*a*) surgical repair commensurate with the endemic incidence rate, and (*b*) for congenital deformities, less than 5% of newly identified cases at or below the recommended age of repair.

 The elimination strategy for each NSD will vary slightly per region but the commonalities will include: case detection at the community level and at birth (for congenital deformities) with subsequent formal referral channels, community door to door outreach by community health workers, advocacy and educational campaigns at all levels, training of the health workforce in screening, case detection, referral, and treatment. This is complemented by partnerships with the local government health department and a partner local collegiate society, NGO, or academic institution who not only works with local institutions and either reimburses cases done but also provides funding and training of the local health workforce in the abovementioned areas.

 Several limitations deserve mention. First, specifying index conditions carry the danger of excluding other conditions that could also be addressed in this type of project; however, as a pilot initiative that seeks validation, a targeted approach renders the logistics around the eradication effort achievable even for an under-resourced country. Subsequent country-led expansion addressing endemic neglected surgical conditions can ensue. Second, disparate measurements of the various NSDs discussed currently exist, necessitating a unified global definition of NSDs centered around eliminating systemic neglect (eg, the mean age of diagnosis of an NSD or the minimum acceptable levels of treatment to prevent disability). This must align with existing initiatives (ie, UHC, eliminating neglected tropical diseases, NTDs) that governments can leverage existing resources to maximize impact. Third, defined surgical lists already exist (eg, Bellwether procedures, DCP3, Amsterdam Declaration on Essential Surgery)^47^ and may presumably add to competing priorities; however, these surgical indices focus on the process of surgical delivery instead of outcomes, which can provide a more accurate picture of the surgical need in remote and underserved areas.

## A Common Definition of Neglected Surgical Diseases

 Defining a subset of neglected surgical conditions that illustrates society’s role and responsibility in addressing them provides a framework through the HRBA lens for its eventual eradication. Strategic guiding principles will be critical in ensuring that we avoid the common pitfalls described above while illustrating the pathway and rationale for adoption. The abovementioned propose a public health framework for surgically correctable conditions that can be identified at the primary care level and subsequently referred and treated at designated institutions with established local partnerships. The framework engages established local public health vehicles and systematizes the solution by targeting disease eradication while strengthening the health system. By providing a new model for disease elimination, the project seeks to create sustainable and resilient surgical infrastructure for the world’s most vulnerable populations while simultaneously providing an incentive for governments to direct investments into strengthening their health systems. Addressing NSDs as a package has the potential to leverage economies of scale in resource-pooling and can represent a ‘best buy’ for governments. We therefore call for the adoption of a common definition for the six index surgical conditions as NSDs to aid in collective implementation of eradication efforts through public health systems strengthening. We believe that this will pave the way for subsequent identification and treatment of other neglected surgical conditions. We will all be best served if we share a common definition of the issue in which we work and to which we encourage others to collaborate towards collective impact to achieve the vision of ending NSDs, achieving surgical integration within UHC, and thus attaining the Sustainable Development Goal vision of ‘Leaving No One Behind’ by 2030.

## Ethical issues

 Ethical approval was obtained from Mount Kenya University Ethics Review Committee (No. MKU/ERC/1167).

## Competing interests

 Authors declare that they have no competing interests.

## Authors’ contributions

 Conception and design: JAH, OH, JB, DH, and RLM. Acquisition of data: ASV, PM, TF, KM, GS, and RO. Analysis and interpretation of data: ASV, JAH, LH, and OH. Drafting of the manuscript: JH, ASV, OH, EA, GS, and RO. Critical revision of the manuscript for important intellectual content: GS, CL, CM, EA, OH, and RL. Obtaining funding: JAH, OH, TF, KM, and PA. Administrative, technical, or material support: KM, TF, SK, EW, ASV, PM, CM, PJ, MN, LN, FA, NP, MLS, RL, EM, DO, AL, and ES. Supervision: PJ, LH, FA, TF, KM, and PM.

## Funding

 This work was supported by the Meru Government of Kenya, Odiongan Municipality of Romblon province, Philippines, and the Henry Family Advised Fund.
